# A Unique Case of Supernumerary Teeth Erupting Inside a Maxillary Sinus Osteoma

**DOI:** 10.3390/jcm13144067

**Published:** 2024-07-11

**Authors:** Toshiyuki Kataoka, Kei Amemiya, Toshiyuki Goto, Hatsuki Kina, Erica Tajima, Toshihiro Okamoto

**Affiliations:** 1 Department of Dentistry and Oral Surgery, Tokyo Women’s Medical University Yachiyo Medical Center, Yachiyo 276-8524, Japan; amemiya.kei@twmu.ac.jp (K.A.); goto.toshiyuki@twmu.ac.jp (T.G.); kina.hatsuki@twmu.ac.jp (H.K.); tajima.erika@twmu.ac.jp (E.T.); 2 Department of Oral and Maxillofacial Surgery, Tokyo Women’s Medical University, Tokyo 162-8666, Japan; okamoto.toshihiro@twmu.ac.jp

**Keywords:** ectopic eruption, supernumerary teeth, osteoma, maxillary sinusitis

## Abstract

**Introduction:** Ectopic foreign bodies in the maxillary sinus occur rarely. Ectopic tooth eruption rarely occurs in the orbit, nasal cavity, maxillary sinus, and elsewhere. Ectopic eruption of teeth in the maxillary sinus is most commonly associated with wisdom teeth and is rarely associated with supernumerary teeth. This rare phenomenon may be accompanied by chronic recurrent sinusitis with headaches and facial pain. However, fibro-osseous lesions in the paranasal sinuses are discovered incidentally on X-ray images and are often asymptomatic. Osteoma is the most common fibro-osseous lesion that develops in the paranasal and nasal sinuses. Osteomas rarely cause serious symptoms such as orbital lesions and intracranial invasion. **Case Presentation:** We report a rare case of exostosis containing supernumerary teeth within the maxillary sinus. A characteristic pedicled bone lesion with a clear border on computed tomography was the undefined orthopantomogram radiopacity in the maxillary sinus, and the lesion contained supernumerary teeth. As the patient had chronic nasal congestion, the tumor was surgically removed. Pathologically, the surgical specimen revealed an osteoma. The patient’s symptoms of chronic sinusitis disappeared. Because the patient had no history of midface trauma or surgery, the supernumerary teeth were speculated to have migrated during a reactive osteogenic process caused by chronic sinusitis. **Conclusions:** A foreign body in the maxillary sinus can be easily diagnosed by computed tomography. Surgical removal is recommended if the foreign body is symptomatic or occupies more than half of the maxillary sinus. This can help resolve chronic sinusitis symptoms and prevent serious complications in the future.

## 1. Introduction

Ectopic eruption of teeth outside the alveolar bone is rare [1]. Reports of ectopic eruption in the nasal septum, mandibular condyle, and maxillary sinus have been reported [2]. Ectopic eruption in the maxillary sinus might be asymptomatic, but could cause symptoms such as chronic or recurrent sinusitis and facial numbness [2]. It has been reported that 1% of patients with chronic sinusitis have ectopic teeth in the maxillary sinus, which might lead to misdiagnosis [3]. However, maxillofacial osteomas occur more frequently in the mandible, with <1% occurring in the midface [4]. In the midface, they occur in the fronto-ethmoid sinus, but are rare in the maxillary sinus [5]. Some reports have revealed enlarged osteomas that have compressed the orbital contents, which led to blindness [6,7] and serious complications such as intracranial invasion [8].

Herein, we report a rare case of ectopic eruption of a supernumerary tooth in an osteoma of the maxillary sinus, and discuss its causes. Nasal congestion due to airway obstruction caused by bony foreign bodies may be resolved by surgical removal of the osteoma or ectopic erupted teeth. It might help in the diagnosis and treatment of chronic sinusitis. Surgical treatment may be selected to prevent serious orbital or intracranial complications.

## 2. Materials and Methods

This case report concerns a 52-year-old man with chronic maxillary sinusitis symptoms, i.e., nasal congestion. Orthopantomography of this patient revealed a well-defined radiopaque in the left maxillary sinus. A CT scan revealed a pedicled bony structure in the left maxillary sinus containing a tooth. The tooth was found to be a supernumerary tooth. The ectopic bony structure was surgically removed. The structure was extracted en bloc via the Caldwell–Luc approach. Histopathological diagnosis was exostosis. The patient’s maxillary sinusitis symptoms disappeared, and he has had no recurrence of symptoms for 2 years. A small number of patients suffering from chronic maxillary sinusitis have ectopic bone lesions in the maxillary sinus. Surgical treatment of ectopic structures in the maxillary sinus appears to be an effective procedure. 

## 3. Case Presentation

**Figure 1:**
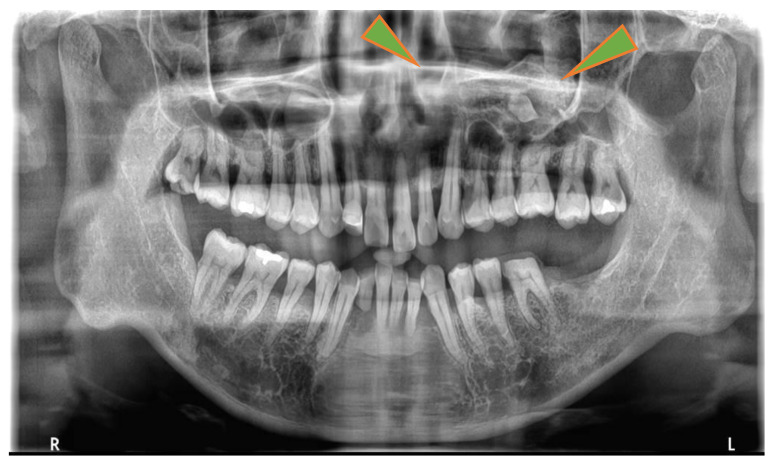
Orthopanoramic radiographs. A well-circumscribed radiopaque is observed in the upper jaw sinus. No permanent tooth deficiency in the maxillary dentition was noted, and wisdom teeth had erupted.

**Figure 2:**
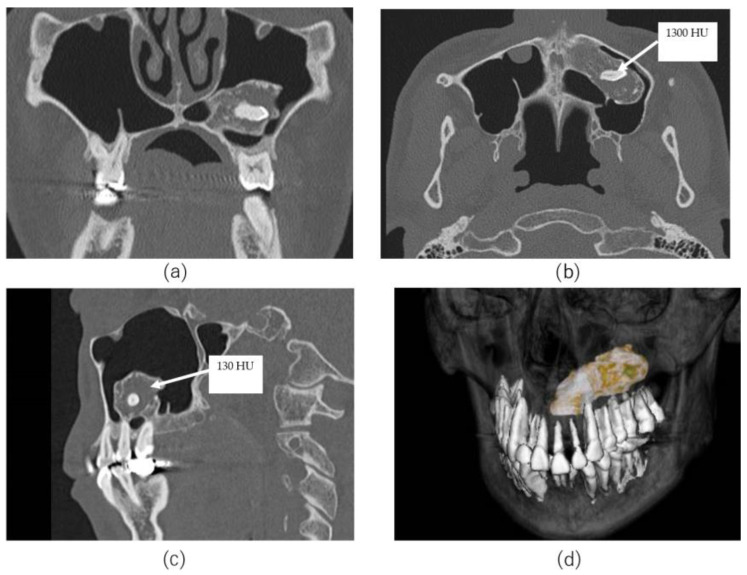
Sectional CT scan and sectional computed tomography. (**a**) Coronal view. Pedunculated mass is depicted from the upper left maxillary sinus. The mass contained a psammoma body and a tooth-like structure. No thickening of the maxillary mucosa was visible. No distortion of the maxillary sinus and no maxillary sinus septa were found. (**b**) Axial view. The mass accounts for approximately half of the left maxillary sinus. The high-intensity radiopacity inside the mass measured 1300 HU. The radiopacity depicts enamel, dentin, and pulp cavity. (**c**) Sagittal view. The mass is continuous with the lower wall of the maxillary sinus. The interior of the tumor showed 130 HU. (**d**) Three-dimensional computed tomography image. The CT value was adjusted to eliminate the low signal range of 1000 HU or less. The surface of the teeth and tumor emerges. The tumor develops within the maxillary sinus to pedunculate from the canine root.

**Figure 3:**
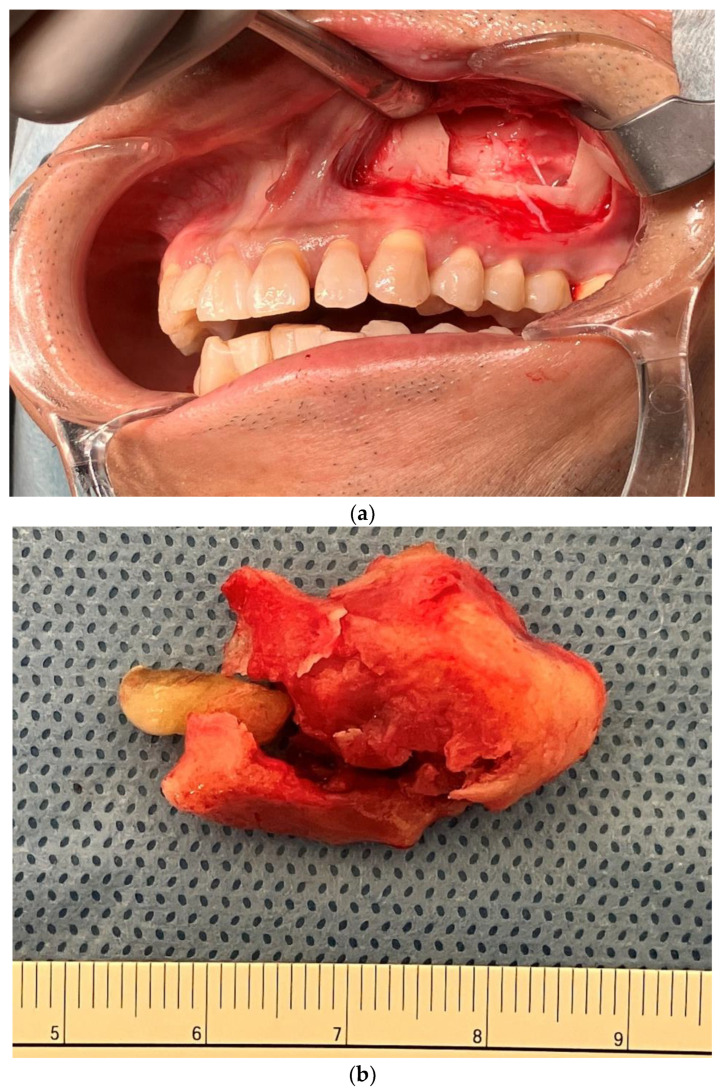
Intraoperative photo and surgical specimen. (**a**) The Caldwell–Luc procedure was performed. The bony window from the anterior wall of the maxillary sinus was formed with piezo-electric surgery. The tumor occupied the maxillary sinus. The tumor surface was bony and sticky. (**b**) Macroscopic appearance of surgical specimen. The tumor was successfully removed en bloc. The tumor contained a supernumerary tooth.

**Figure 4:**
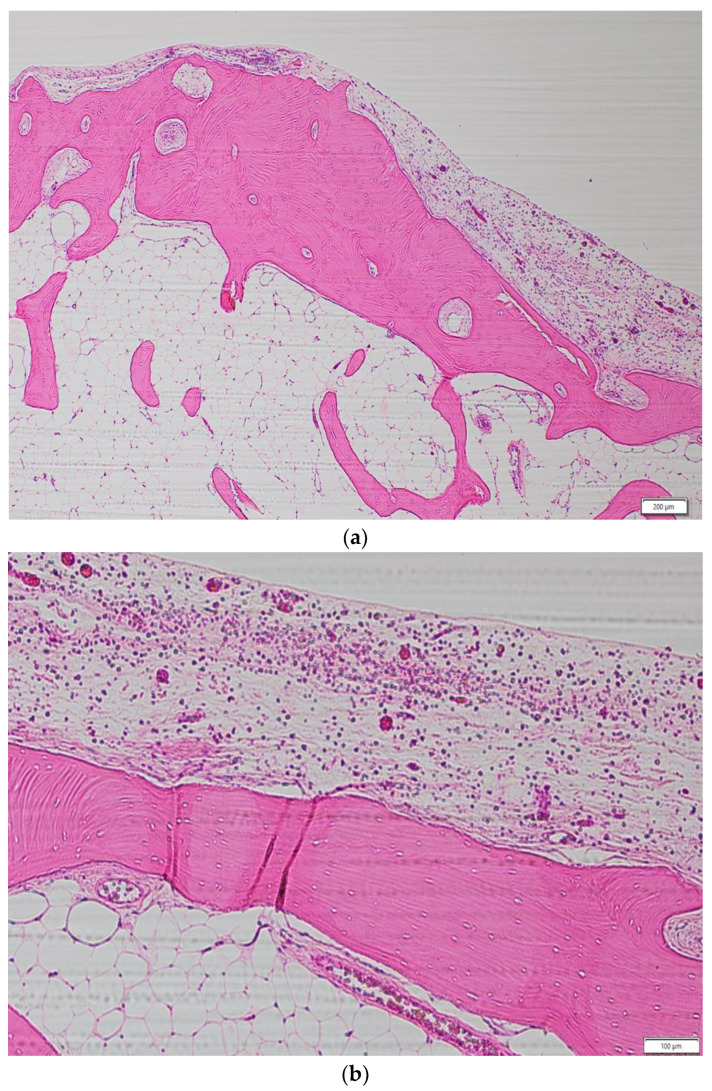
Image of pathological photograph (hematoxylin and eosin-stained). (**a**) A view of lesion at ×4 magnification. The specimen revealed a mixture of mature lamellar bone and various sizes of Haversian lamellae under the fibrous capsule. (**b**) A view of lesion at ×10 magnification. Fatty marrow was observed between the trabeculae. No inflammatory cell infiltration and epithelial odontogenic cells were noted.

A 52-year-old man presented for evaluation of pantomogram radiopacity in the maxillary sinus. He had no history of systemic diseases. He had chronic nasal congestion and was diagnosed with chronic sinusitis by an otolaryngologist; however, no treatment was given. On intraoral examination, the wisdom teeth had erupted, and no teeth were missing. He felt a slight percussion pain in his upper left second premolar, first molar, and second molar; however, tooth mobility was not observed, and all teeth were vital. No bulge in the alveolar region and no rhinorrhea were noted. He had no history of midfacial trauma or surgery, and the patient recalled not receiving orthodontic treatment. An orthopantomographic X-ray image showed a well-defined radiopacity in the left maxillary sinus, which contained a psammoma body and a tooth or an odontoma-like structure. The contralateral maxillary sinus was clear, and all permanent teeth had not received root canal treatment (Figure 1). A sectional CT view showed a bony mass at the bottom of the left maxillary sinus. The pedunculated mass on the lower wall of the maxillary sinus measured 37 × 21 × 24 mm. This mass was qualitatively examined using Hounsfield units (HU). The center of the mass was 130 HU, which was equivalent to cancellous bone, and the tooth-like structure component with enamel, dentin and pulp measured 1300 HU (Figure 2). The clinical diagnosis was a foreign body in the maxillary sinus, i.e., osteoma, or an odontogenic tumor containing an impacted tooth. Under general anesthesia, the tumor was removed from the left maxillary sinus using the Caldwell–Luc approach. After the bony window from the anterior wall of the maxillary sinus was lifted, the osseous tumor was visible. The tumor stalk was resected using piezosurgery and removed en bloc. As his osteomeatal complex was not obstructed, an inferior meatal antrostomy was not performed. No bleeding in the maxillary sinus mucosa was observed after tumor removal (Figure 3). Histopathologically, the surgical specimen was composed of mature lamellar bone under a fibrous capsule and fatty marrow between the trabeculae. Inflammatory cells and odontogenic epithelium were not observed (Figure 4). The final pathological diagnosis was osteoma, exostosis. The patient’s nasal congestion resolved, and he remained symptom-free 2 years after the surgery.

## 4. Discussion

Osteoma is a benign and slow-growing tumor. Osteomas are thought to emerge from the embryogenic periosteum [9], and the annual growth rate is 0.79 mm/year on average [10]. Suggested causes of osteomas include trauma, infection, and inflammation; however, the exact etiology is still unclear [11,12]. Among the craniofacial bones, it frequently occurs in the mandible, with a ratio of 7:3 [13]. In the paranasal sinus, osteomas occur in 0.01%–0.43% of patients [5], predominantly in the frontal sinus [6,14]. Osteomas occurring in the maxillary sinus have been rarely reported [5,9]. Osteomas of the maxillary sinus are discovered incidentally in orthopantomography during screening in a dental practice. Common clinical symptoms include headache and facial pain [11]. If paranasal sinus osteomas are asymptomatic or when the mass lesion occupies 50% or less of the sinus volume, it be observed with regular radiological examinations rather than with surgical intervention [15]. If multiple osteomas are found in the midface, they must be differentiated from malignant tumors such as osteosarcoma and Gardner’s syndrome (GS) [16]. In cases of multiple osteomas of the craniofacial bones, extraintestinal manifestations of GS that precede polyposis formation are suspected [16]. Large tumors that occupy the sinuses can cause diplopia, epiphora, and blindness [7,17].

Ectopic teeth and supernumerary teeth are found in approximately 1% of the total population [18]. Dentists frequently find ectopic tooth eruptions within the dental structure. Those occurring in nondental areas, such as the mandibular condyle, coronoid process, orbit palate, and nasal cavity, have been rarely reported [19]. In a literature review of ectopic eruption, the maxillary sinus was the most frequently involved area [20]. Ectopic tooth development and eruption may involve multifactorial pathological disorders with both general and local etiological factors, or their combination [21,22]. These include maxillofacial trauma, odontogenic or rhinogenic infections, developmental anomalies such as cleft palate, genetic factors such as Turner syndrome and Apert syndrome [23,24], and jawbone lesions such as odontogenic cysts and tumors [25]. Local factors include maxillary hypoplasia, retrognathism, and crowding [3,26]. The prevalence of ectopic teeth in the maxillary sinus is the highest in the third molars [25], and supernumerary teeth are rare [27]. Ectopic eruption in the maxillary sinus may be symptomatic. Symptoms often include nasal congestion, facial fullness, headache, and facial numbness [2]. In addition, 1% of patients provisionally diagnosed with chronic sinusitis have ectopic teeth in the maxillary sinus [3]. In addition, approximately 80% of ectopic teeth have been removed [2]. A common treatment for ectopic teeth in the maxillary sinus is surgical removal using the Caldwell–Luc approach [28]. Regular examination with orthopantomography or CT is preferred in asymptomatic cases because they may go on to form cysts and tumors [27,29].

This presented case is probably the first case in which a supernumerary tooth erupted within the maxillary sinus osteoma. In a similar case of osteoma in the maxillary sinus, an ectopic third molar coexisted independently [30]. In the present case, the patient had no history of surgery involving the maxillary sinus or midface trauma. Nasal congestion symptoms were diagnosed by an otolaryngologist as chronic sinusitis; however, he did not notice the ectopic teeth because he had not undergone CT of the maxillary sinus. The surgical and histopathological findings of this case did not indicate endoostosis such as fibrous dysplasia, insertion of bone fragments because of previous surgery or trauma, or new bone formation. The tumor, which had a unique morphology with a pedicled, arcuate appearance and a histological composition in which the cortical bone on the surface of the tumor covered the fatty marrow inside, was histologically not a septum of a sinus, which is composed of bone cortex. Tooth eruption changes the vertical level of the bone, but does not completely cover the crown of the tooth, like a dentigerous cyst. Therefore, no researchers have theorized that an impacted tooth induces the bone to form an osteoma around the tooth. The primary cause of osteoma formation in the maxillary sinus remains unknown; however, some reports suggest that chronic inflammatory stimulation may contribute to osteoma formation [31,32,33]. We speculate that the pathogenesis of this rare entity is the accidental migration of supernumerary teeth into a reactive osteogenic process caused by chronic inflammation. If the osteomeatal complex is obstructed by a foreign body within the maxillary sinus (osteomatous lesions or teeth), chronic sinusitis may occur [3], and if it occupies >50% of the sinus volume, surgical treatment is required [15], i.e., resection by the Caldwell–Luc approach [28], particularly if the mass is located in the lower part of the maxillary sinus. The foreign body located in the upper part of the maxillary sinus may be surgically operated endoscopically [34]. When removing large lesions, postoperative complications, such as aesthetic and functional deficits, must be considered [35]. When applying for surgery, the surrounding anatomical structures must be preserved as much as possible, and we believe that a combined endoscopic [36] (functional endoscopic sinus surgery) and intraoral approach is better [37]. Regular X-ray examinations are recommended if the bone heterotopic foreign body in the maxillary sinus is asymptomatic or occupies <50% of the maxillary sinus. Malignancy should always be suspected in large tumors or those with destructive infiltration into the surrounding area. In cases of multiple lesions of the craniofacial bones, systemic diseases such as G.S. must be differentiated. If it is more inconvenient or the osteomeatal complex is obstructed, surgery may be advisable to prevent serious complications.

## 5. Conclusions

We report a case of chronic maxillary sinusitis with ectopic exostosis in the maxillary sinus in which nasal congestion symptoms improved after surgery. In some patients who complain of sinusitis, a foreign body in the maxillary sinus is misdiagnosed as sinusitis. A detailed otorhinolaryngological examination and a specialized dental examination by a general dentist (including evaluation of lesions such as third molars, supernumerary teeth, growth deficiency, dental crowding, and odontogenic tumors) will help determine an appropriate treatment plan. Dental cone beam computed tomography is a simple and detailed examination to detect impacted teeth and ectopic structures. Surgical intervention is recommended in symptomatic cases with ectopic eruption of teeth and ectopic foreign bodies in the maxillary sinus. Surgical management prevents serious complications that may occur from large osteomas, such as orbital lesions and intracranial infiltration. The exact cause of maxillary sinus osteoma formation remains unknown, and further accumulation and analysis of various cases is needed.

## Data Availability

The data that support the findings of this study are available upon request from the corresponding author. The data are not publicly available, due to privacy or ethical restrictions.
